# A Peculiar Case of Rapidly Recurring Metastasis of Malignant Non-small Cell Primary Lung Carcinoma to the Heart

**DOI:** 10.7759/cureus.58009

**Published:** 2024-04-10

**Authors:** Geoff J Thomas, Vivie Tran, Anthony Pham, Ardalan Naghian, Mohammad M Ansari

**Affiliations:** 1 Cardiology, Texas Tech University Health Sciences Center, Lubbock, USA

**Keywords:** metastatic lung cancer, malignant tumor resection, intracardiac tumor, right atrial thrombus, right atrial cardiac mass

## Abstract

We report the case of a 64-year-old adult male with a rapidly recurring metastatic lung carcinoma in the right atrium of the heart. Advanced-stage lung carcinomas can metastasize to other organs such as the heart, bones, brain, liver, adrenal glands, and lymphatic system, although actual rates of metastasis to the heart are relatively quite low. This patient was diagnosed with a right atrial mass that was determined through pathology to be a result of an existing non-small cell lung carcinoma. This mass, despite resection, reappeared two weeks later at the same location and with a similar size to the previous metastatic tumor. This case highlights the importance of closely monitoring sites of resected tumors for potential regrowth and complications.

## Introduction

Intracardiac masses can arise as a result of either thrombosis or the formation of tumors within the heart. These masses often pose a diagnostic challenge due to their nonspecific symptoms, which can mimic other heart diseases. Consequently, they frequently go unnoticed until after the patient's death [1]. To aid in the diagnosis of these masses, various imaging modalities are available, including transthoracic echocardiography (TTE) and transesophageal echocardiography (TEE) [2].

Cardiac tumors are considered rare, with a frequency range that is less than 0.33% [3]. The majority of these tumors are metastatic in nature, meaning they originate from cancer that has spread to the heart either directly or through the bloodstream, lymphatic system, or by diffusion through the inferior vena cava or pulmonary veins [4]. Cardiac metastases can have diverse origins, with approximately 36% to 39% arising from lung cancer, 10% to 21% originating from hematologic malignancies, and 10% to 12% originating from breast cancer [5].

Here, we present a rare case involving a 64-year-old male patient who was found to have a recurring mass in the right atrium of his heart during imaging studies.

## Case presentation

This patient is a male aged 64 years with a past medical history of cerebrovascular accident (CVA) with a left middle cerebral artery stroke, a left internal carotid artery occlusion, mural aortic thrombus, hypertension (HTN), tobacco abuse for the past 30 years, chronic obstructive pulmonary disease (COPD), and lung nodules presenting with shortness of breath and elevated troponin concerning for non-ST-elevation myocardial infarction (NSTEMI). Selective coronary angiography, echocardiography, and computed tomography (CT) were performed on this patient.

**Figure 1:**
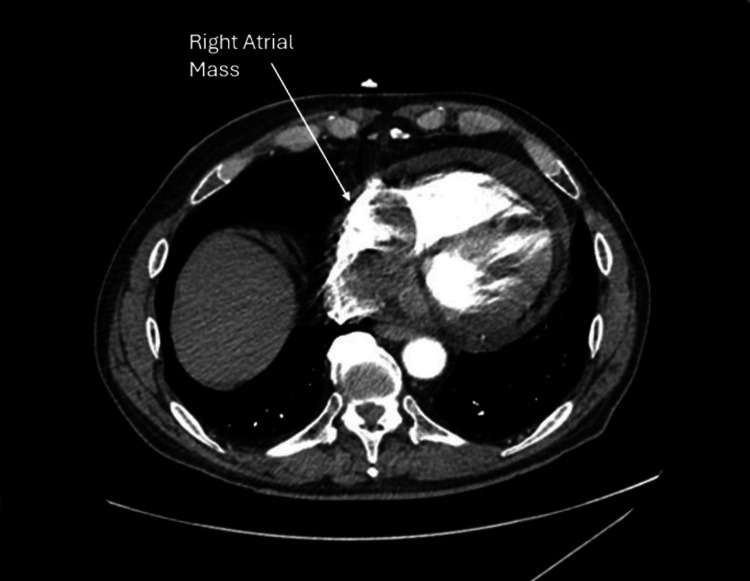
CT image of the heart showing the right atrial mass CT: Computed tomography

**Figure 2:**
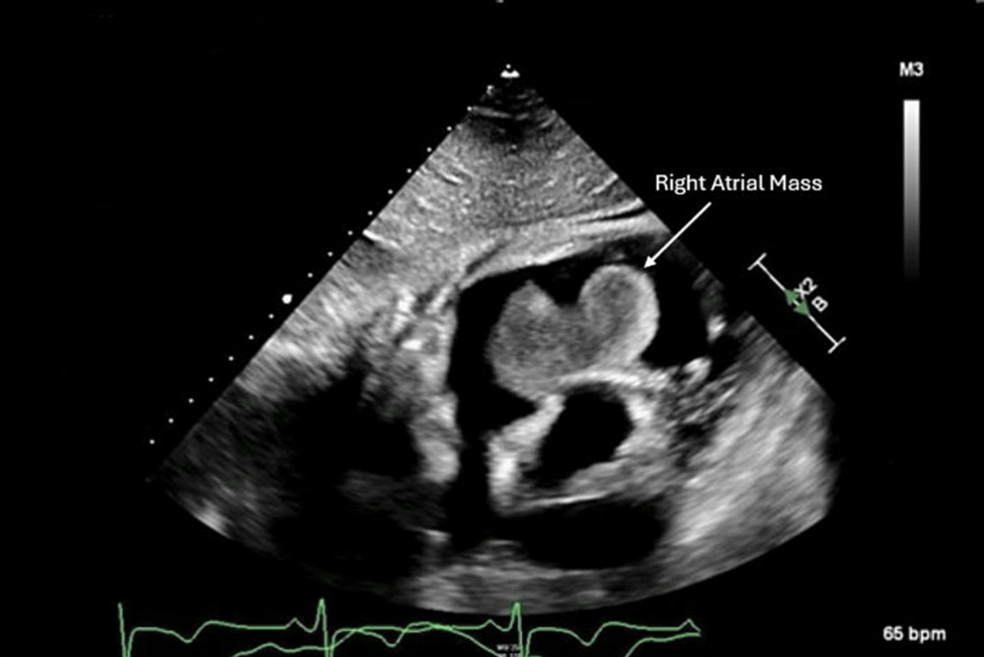
Echocardiography showing a pedunculated right atrial mass

Coronary angiography showed a three-vessel coronary artery disease (CAD). Imaging from echocardiography and CT revealed a 7.1 cm x 3.1 cm pedunculated right atrial mass (Figures 1-2). Initially, the patient underwent percutaneous clot retraction with little success as the mass was still visible at the location, after which the patient underwent surgical extraction of the mass which was successfully performed.

**Figure 3:**
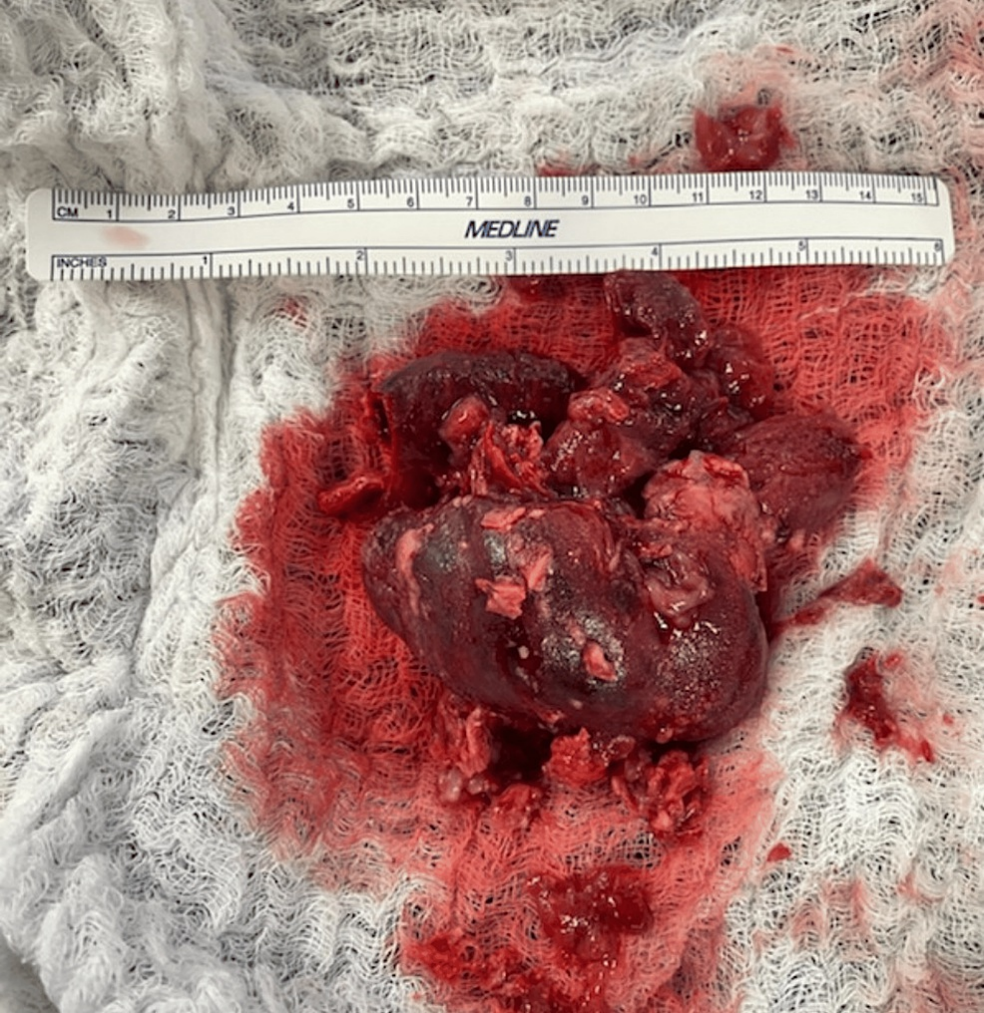
Image of a surgically resected right atrial mass

The 7.1 cm x 3.1 cm mass was visualized within the right atrium and was completely resected (Figure 3). The mass was found to be extending down into a part of the right ventricle as well as into the coronary sinus, causing minor blockage of the coronary sinus. The entirety of the mass as well as the surrounding area was declotted and debulked as much as possible. Furthermore, the left atrial appendage was ligated due to concerns for future thrombi formation. Upon initial impressions, it appeared to be an old clot, but due to uncertainty, the mass was sent to Mayo Clinic Laboratories for pathology.

Histology of the sample revealed a poorly circumscribed mass composed of large malignant epithelioid cells. There was also marked nuclear pleomorphism with coarse chromatin, prominent nucleoli, multinucleation, frequent mitosis, and multifocal tumor necrosis. These findings, combined with several immunohistochemistry stains, determined that the mass was a metastasis from a primary lung carcinoma.

**Figure 4:**
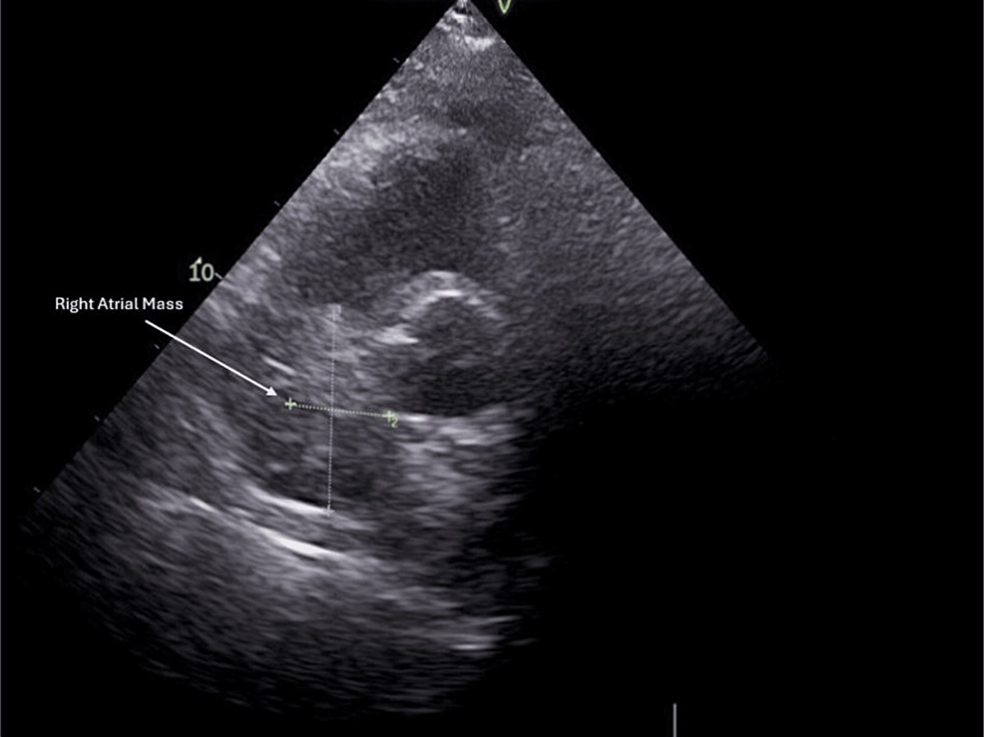
Echocardiography showing a recurrent right atrial mass two weeks after surgical resection of the initial mass

Patient had a relatively uneventful postoperative course and was subsequently discharged only to return two weeks later with repeat imaging revealing a new malignancy of similar size at 6.8 cm x 2.7 cm presenting in the exact same location as the initial mass (Figure 4). The repeat echocardiogram of the patient done at another facility confirmed by two separate physicians confirms the recurrence of the mass.

## Discussion

Metastasis is the leading cause of death in lung cancer patients [6]. The most common sites for lung cancer metastasis include the lymph nodes, liver, brain, bones, and adrenal glands [1]. However, the occurrence of metastasis to the heart varies depending on the specific type of lung cancer. Adenocarcinoma accounts for 26% of cases, squamous cell carcinoma for 23.4%, undifferentiated carcinoma for 21.2%, and bronchoalveolar carcinoma for 17.4% [7]. Cardiac metastasis often remains silent and is typically only diagnosed during autopsy in around 90% of patients [1]. Clinical symptoms arise due to various factors, such as the obstruction of cardiac or valve function, interference with coronary blood flow, disruptions to the heart's electrical activity, and the presence of pericardial effusion. Possible symptoms include chest pain, dyspnea, edema of the lower extremities, and new-onset atria fibrillation and can progress to life-threatening conditions such as myocardial infarction (MI) and heart block [8].

The utilization and widespread availability of advanced imaging techniques have greatly enhanced the incidental identification of cardiac metastasis. Echocardiography is the preferred initial method for detecting and diagnosing cardiac metastases [9]. When compared to TTE, CT, magnetic resonance imaging (MRI), and angiography, TEE offers superior visualization of the atria and major vessels. CT and MRI are valuable imaging modalities for evaluating cardiac metastasis as they provide detailed information regarding its location, shape, size, local invasion, and potential involvement of the mediastinum or lungs. Moreover, these techniques can offer insights into the histological characteristics of metastatic lesions, such as the presence of fat, calcification, fibrous tissue, melanin, hemorrhage, or cystic changes. Contrast agents are often utilized in conjunction with these imaging modalities to aid in distinguishing between tumors and blood clots [10].

There have been some case studies reported in the literature highlighting instances of lung cancer metastasizing to the heart. For example, Alexandrescu et al. documented a case in 2009 where adenocarcinoma originating from the right lower lobe of the lung metastasized to the right atrium [11]. Similarly, Prasad et al. described a case in 2016 involving a patient with an unknown primary adenocarcinoma that had spread to the left side of the heart [12]. However, none showed a reoccurrence within two weeks, making our case the first reported case in the literature of such a rapid recurrence.

In contrast to the other published cases, our case demonstrates the first reported occurrence of rapidly recurring metastasis (within two weeks) from a malignant non-small cell lung carcinoma to the heart. While metastasis of a primary lung carcinoma to the heart has been reported in the past, our case demonstrates an exceptional situation in which the cardiac metastatic tumor reappeared in the exact same spot and grew back to almost 7 cm at an exceptionally fast rate in a matter of two weeks. It is also important to note that this reappearance occurred despite a thorough and successful procedure to declot and debulk the tumor in its entirety as well as the surrounding regions in the heart that were also affected. This was confirmed by the two surgeons present in the procedure and reconfirmed by the follow-up imaging prior to the initial discharge. This further demonstrates how exceptional the growth of this cardiac tumor is, as its reappearance appears to have occurred on its own rather than the result of an improperly resected mass.

## Conclusions

Our case of a malignant non-small cell primary lung carcinoma to the heart appears to be the first reported instance of a metastatic cardiac tumor reappearing in the same location and with a similar size just two weeks after resection of the initial tumor. In clinical settings, the main emphasis is usually placed on the diagnosis and treatment of lung cancer, which can result in the overlooking of cardiac metastasis originating from advanced stages of the disease. Cardiac metastasis serves as a significant indicator of cancer that has extensively spread throughout the body, often associated with a poor prognosis. This case highlights the importance of rapid and consistent monitoring for metastatic cardiac tumors in those with lung cancer, even after initial treatments and surgery.
